# CKD-EPI and Cockcroft-Gault Equations Identify Similar Candidates for Neoadjuvant Chemotherapy in Muscle-Invasive Bladder Cancer

**DOI:** 10.1371/journal.pone.0094471

**Published:** 2014-04-10

**Authors:** Sumanta K. Pal, Nora Ruel, Sergio Villegas, Mark Chang, Kara DeWalt, Timothy G. Wilson, Nicholas J. Vogelzang, Bertram E. Yuh

**Affiliations:** 1 Department of Medical Oncology and Experimental Therapeutics, City of Hope Comprehensive Cancer Center, Duarte, California, United States of America; 2 Division of Biostatistics, Department of Information Science, City of Hope Comprehensive Cancer Center, Duarte, California, United States of America; 3 Division of Urology, Department of Surgery, City of Hope Comprehensive Cancer Center, Duarte, California, United States of America; 4 US Oncology Research, Comprehensive Cancer Centers, Las Vegas, Nevada, United States of America; Eberhard-Karls University, Germany

## Abstract

Clinical guidelines suggest neoadjuvant cisplatin-based chemotherapy prior to cystectomy in the setting of muscle-invasive bladder cancer (MIBC). A creatinine clearance (CrCl) >60 mL/min is frequently used to characterize cisplatin-eligible patients, and use of the CKD-EPI equation to estimate CrCl has been advocated. From a prospectively maintained institutional database, patients with MIBC who received cystectomy were identified and clinicopathologic information was ascertained. CrCl prior to surgery was computed using three equations: (1) Cockcroft-Gault (CG), (2) CKD-EPI, and (3) MDRD. The primary objective was to determine if the CG and CKD-EPI equations identified a different proportion of patients who were cisplatin-eligible, based on an estimated CrCl of >60 mL/min. Cisplatin-eligibility was also assessed in subsets based on age, CCI score and race. Actuarial rates of neoadjuvant cisplatin-based chemotherapy use were also reported. Of 126 patients, 70% and 71% of patients were found to be cisplatin-eligible by the CKD-EPI and CG equations, respectively (P = 0.9). The MDRD did not result in significantly different characterization of cisplatin-eligibility as compared to the CKD-EPI and CG equations. In the subset of patients age >80, the CKD-EPI equation identified a much smaller proportion of cisplatin-eligible patients (25%) as compared to the CG equation (50%) or the MDRD equation (63%). Only 34 patients (27%) received neoadjuvant cisplatin-based chemotherapy. Of the 92 patients who did not receive neoadjuvant chemotherapy, 64% had a CrCl >60 mL/min by CG. In contrast to previous reports, the CKD-EPI equation does not appear to characterize a broader span of patients as cisplatin-eligible. Older patients (age >80) may less frequently be characterized as cisplatin-eligible by CKD-EPI. The discordance between actual rates of neoadjuvant chemotherapy use and rates of cisplatin eligibility suggest that other factors (e.g., patient and physician preference) may guide clinical decision-making.

## Introduction

Several therapeutic options are available to patients with muscle-invasive bladder cancer (MIBC). For patients who opt to receive radical cystectomy, current guidelines strongly recommend consideration of neoadjuvant cisplatin-based chemotherapy.[1], [2] These guidelines are predicated on randomized, phase III trials showing a survival benefit with this modality.[3], [4] For instance, in Southwest Oncology Group (SWOG) 8710, a total of 307 patients with MIBC were randomized to receive either methotrexate, vinblastine, adriamycin and cisplatin (MVAC) followed by cystectomy or cystectomy alone.[4] Median survival was improved in those patients who received neoadjuvant MVAC (77 months *v* 46 months, P = 0.06). Alternatives to neoadjuvant MVAC include dose-dense MVAC (ddMVAC; supported by prospective phase II data), gemcitabine-cisplatin (GC; supported by retrospective series) and cisplatin/methotrexate/vinblastine (CMV; supported by a recently published phase III trial).[3], [5], [6] Meta-analytic data pooled across multiple studies of neoadjuvant cisplatin-based trials suggest an absolute 5%.[7] From a theoretical perspective, the efficacy of neoadjuvant therapy may be based on (1) cytoreduction of primary tumor, resulting in more complete and successful surgery, or (2) elimination of micrometastatic disease burden.

Common to present neoadjuvant chemotherapy regimens for MIBC is the inclusion of cisplatin chemotherapy. Cisplatin has been used for the systemic management of bladder cancer for over three decades, and the nephrotoxicity associated with it has been well documented.[8], [9] In light of this, clinical trials evaluating cisplatin-based regimens frequently include cutoffs for appropriate renal function. A common threshold in current day clinical trials is a creatinine clearance (CrCl) of 60 mL/min, below which patients are deemed ineligible.[10] These thresholds vary, with other groups utilizing thresholds of 45 and 55 mL/min to characterize eligibility for cisplatin-based studies.[11], [12] Although it has been suggested that a measured CrCl is ideal, several studies allow for a calculated CrCl.

Methods for calculation of estimated CrCl vary. Although the Cockcroft-Gault equation has traditionally been employed, the reduced accuracy of this equation in certain populations (i.e., older adults) has long been recognized.[13] Alternatives to the Cockroft-Gault equation include the Modified Diet in Renal Disease (MDRD) equation, developed from a training cohort of 1,070 patients, and validated in a cohort of 558 patients.[14] At the time this equation was conceived, it was thought to be more accurate than measured CrCl. More recently, in 2009, a new equation was introduced by the Chronic Kidney Disease Epidemiology (CKD-EPI) collaboration.[15] The eponymously titled CKD-EPI equation was suggested to offer a more precise assessment of glomerular filtration as compared to previous equations. Tsao *et al* have recently examined the Cockcroft-Gault and CKD-EPI equations in a series of 116 patients with bladder cancer treated at a single institution.[16] The authors reported that patients were 17% more likely to be deemed ineligible for cisplatin-based chemotherapy when using the Cockcroft-Gault equation as compared to the CKD-EPI equation, using a cutoff of 60 mL/min. In the current manuscript, we sought to validate these findings by comparing the two equations in an independent patient series. Our study captures a more extensive array of relevant clinical variables, such as comorbidity and actuarial use of chemotherapy, in a series of patients with muscle-invasive bladder cancer (MIBC) who received definitive management with cystectomy.

## Patients and Methods

### Patients

Patient data was obtained from an institutional bladder cancer database through the City of Hope Institutional Review Board approved protocol (IRB 12131). This database has been prospectively maintained from 1995 onwards with de-identified patient information. Prior to 2003, cases were primarily performed using a traditional open approach, whereas subsequent to 2003, cases were performed using robotic-assisted laparoscopic techniques. The database contains extensive clinical and demographic data, including age, race, gender, and Charlson comorbidity index (CCI). CCI is a system that assigns points for comorbidities and when added together can be used to predict mortality. Patients were included in the current study if they were noted to have muscle-invasive urothelial carcinoma of the bladder (other histologies, such as small cell and adenocarcinoma, were excluded. The nature and duration of neoadjuvant chemotherapy is also available within the database. Importantly, renal function and body-mass index (BMI) are recorded at the time of the initial patient encounter (i.e., prior to receipt of neoadjuvant chemotherapy and surgical intervention). These values were used to calculate CrCl using the subsequently defined methodology.

### Calculation of CrCl

Standard methods were used to generate the estimates of CrCl using the Cockroft-Gault, CKD-EPI, and MDRD equations. Specifically, the Cockcroft-Gault equation used was as follows:
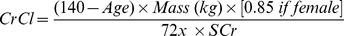
Notably, CrCl and SCr refer to CrCl and serum creatinine, respectively. The CDK-EPI equation used was as follows:
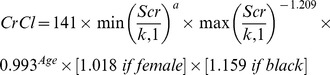
The MDRD equation used was as follows:
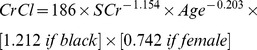



### Statistical Analysis

Demographic, clinicopathologic information and clinical outcomes data were reported, with subgroups based on actuarial receipt of neoadjuvant cisplatin-based chemotherapy. The primary endpoint of this retrospective analysis was to determine if the Cockroft-Gault and CKD-EPI equations identified different proportions of cisplatin-eligible patients amongst patients with MIBC who received cystectomy. Cisplatin eligibility was defined as a CrCl of 60 mL/min. Effect of calculation method on eligibility proportions was compared using a one way ANOVA test. Testing for differences in demographic or clinicopathologic variables between patient subgroups (Tables 1 and 2) was done using the chi-square test and student's *t*-test for categorical and continuous data, respectively. As a secondary endpoint, the proportion of patients deemed cisplatin-eligible by the MDRD equation was compared to the proportion derived from the CKD-EPI equation. Cisplatin-eligibility was also assessed in subsets based on age, CCI score and race.

**Table 1:** Demographic and clinicopathologic characteristics, as well as selected clinical outcomes, of patients with MIBC who received radical cystectomy.

	All Patients (n = 126)	(1) Chemo+ CG< = 60 (n = 5)	(2) Chemo+ CG>60 (n = 29)	(3) Chemo- CG< = 60 (n = 33)	(4) Chemo- CG>60 (n = 59)	p-value
Gender, n (%)						
Female	21 (16.7%)	1 (20.0%)	2 (6.9%)	10 (27.8%)	8 (14.3%)	0.1
Male	105 (83.3%)	4 (80.0%)	27 (93.1%)	23 (69.7%)	51 (86.4%)	
Surgery Age, median (IQR)	71.5 (64–78)	77 (73–85)	65 (59–69)	79 (77–83)	69 (62–73)	<0.0001
BMI, median (IQR)	26.8 (24.1–30.9)	22.9 (21.0–29.0)	29.4 (26.7–33.3)	24.3 (23.4–25.9)	27.7 (24.7–32.2)	0.002
ASA, n (%)						
II	24 (19.0%)	2 (40.0%)	4 (13.8%)	3 (9.1%)	15 (25.4%)	0.2
III	80 (63.5%)	1 (20.0%)	21 (72.4%)	23 (69.7%)	35 (59.3%)	
IV	22 (17.5%)	2 (40.0%)	4 (13.8%)	7 (21.2%)	9 (15.3%)	
Total CCI, median (IQR)	5 (3–8)	6 (4–8)	5 (3–8)	8 (4–9)	4 (2–8)	0.2
Clinical T Stage, n (%)						
T2	117 (92.9%)	4 (80.0%)	26 (89.7%)	31 (93.9%)	56 (94.9%)	0.5
T3	6 (4.8%)	1 (20.0%)	2 (6.9%)	2 (6.1%)	1 (1.7%)	
T4	3 (2.4%)	0 (0.0%)	1 (3.4%)	0 (0.0%)	2 (3.4%)	

Subgroups are based on (1) receipt or non-receipt of neoadjuvant cisplatin-based chemotherapy and (2) CrCl (above or below 60) based on the Cockroft-Gault (CG) equation.

**Table 2:** Surgical outcomes and pathologic findings of patients with MIBC who received radical cystectomy.

	All Patients (n = 126)	(1) Chemo+ CG< = 60 (n = 5)	(2) Chemo+ CG>60 (n = 29)	(3) Chemo- CG< = 60 (n = 33)	(4) Chemo- CG>60 (n = 59)	p-value
Diversion Type, n (%)						
Ileal Conduit	44 (34.9%)	3 (60.0%)	5 (17.2%)	17 (51.5%)	19 (32.2%)	0.005
Indiana Pouch	31 (24.6%)	1 (20.0%)	4 (13.8%)	10 (30.3%)	16 (27.1%)	
Studer neobladder	51 (40.5%)	1 (20.0%)	20 (69.0%)	6 (18.2%)	24 (40.7%)	
Surgery Length, hours median (IQR)	7.2 (6.3–8.4)	7.6 (7.3–8.1)	7.5 (6.6–8.3)	6.7 (5.9–7.8)	7.2 (6.4–8.8)	0.2
EBL, ml median (IQR)	400 (250–550)	500 (200–700)	350 (250–500)	350 (225–525)	400 (250–550)	0.4
Pathologic Stage, n (%)						
<T2	30 (23.8%)	1 (20.0%)	14 (48.3%)	2 (6.1%)	13 (22.0%)	0.03
T2	43 (34.1%)	1 (20.0%)	6 (20.7%)	12 (36.4%)	24 (40.7%)	
T3	39 (31.0%)	2 (40.0%)	6 (20.7%)	15 (45.4%)	16 (27.41%)	
T4	14 (11.1%)	1 (20.0%)	3 (10.3%)	4 (12.1%)	6 (10.2%)	
Pathologic Node Status, n (%)						
N0	89 (70.6%)	3 (60.0%)	17 (58.6%)	23 (69.7%)	46 (78.0%)	0.1
N1	11 (8.7%)	1 (20.0%)	5 (17.2%)	3 (9.1%)	2 (3.4%)	
N2	22 (17.5%)	0 (0.0%)	6 (20.7%)	6 (18.2%)	10 (16.9%)	
N3	1 (0.8%)	0 (0.0%)	0 (0.0%)	1 (3.0%)	0 (0.0%)	
NX	3 (2.4%)	1 (20.0%)	1 (3.4%)	0 (0.0%)	1 (178%)	
Length of Stay, days median (IQR)	9.5 (7–14)	10 (9–13)	9 (7–14)	11 (8–15)	9 (7–14)	0.4

Subgroups are based on (1) receipt or non-receipt of neoadjuvant cisplatin-based chemotherapy and (2) CrCl (above or below 60) based on the Cockroft-Gault (CG) equation.

## Results

### Patient Characteristics

A total of 126 patients were identified who had (1) a diagnosis of MIBC, (2) had received cystectomy at City of Hope, and (3) had sufficient data available to assess renal function by the three proposed methods. The majority of patients were male (83%). A total of 34 patients (27%) received neoadjuvant chemotherapy. Of these 34 patients, 29 patients (85%) had a CrCl >60 mL/min. Interestingly, amongst patients who did not receive chemotherapy (n = 92), the majority (64%) also had a CrCl >60 mL/min. As noted in Table 1, median age was higher in those patients with a CrCl ≤60 mL/min, and median body-mass index (BMI) was lower in these cohorts, as well. Few patients (<10%) were characterized as having clinical T3 or T4 disease.

### Surgical Outcome and Pathologic Findings

As summarized in Table 2, Studer orthotopic neobladder, ileal conduit, and Indiana pouch urinary diversions were performed with similar frequency (40%, 35%, and 25% respectively). As it is our practice to avoid continent diversions in patients with reduced renal function, continent diversions (Indiana Pouch or Studer neobladder) were more frequent in patients with higher CrCl. Median operative time and estimated blood loss did not vary significantly amongst groups subdivided by chemotherapy receipt and Cockcroft-Gault calculated CrCl. Despite the fact that <10% of patients were characterized as having clinical T3/T4 disease prior to cystectomy, 31% were characterized as having pathologic T3 disease and 11% were characterized as having pathologic T4 disease. The majority of patients with pathologic T3 and T4 disease had not received neoadjuvant chemotherapy, while the majority of patients with pathologic down-staging (i.e., findings of pathologic T0, Ta or T1 disease) had received this modality.

### Cisplatin Eligibility

**Figure 1:**
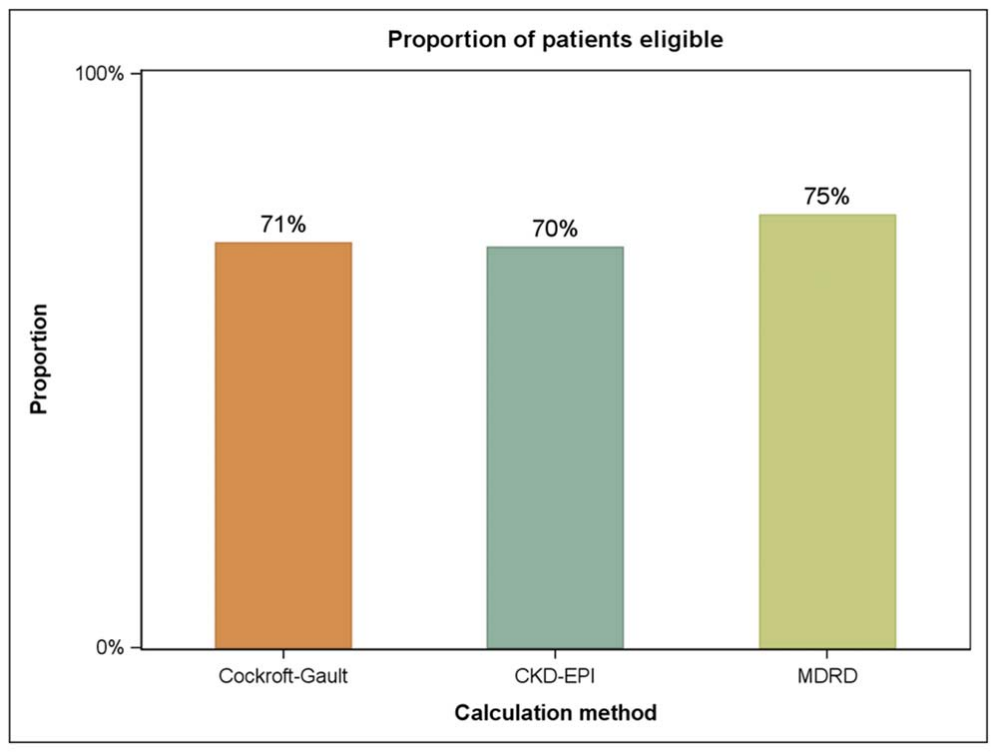
Proportion of patients defined as cisplatin-ineligible (i.e., creatinine clearance >60) based on the CKD-EPI equation, Cockroft-Gault equation, and MDRD equation (n = 126).

As previously noted, cisplatin eligibility was defined by a calculated CrCl of >60 mL/min. The primary objective of the study was to determine if the CKD-EPI equation identified a greater proportion of patients to be cisplatin-eligible as compared to the Cockcroft-Gault equation, to support previously published studies.[16] Ultimately, as noted in Figure 1, there was no difference in this proportion, with 70% and 71% of patients deemed cisplatin-eligible by the CKD-EPI and Cockcroft-Gault methods, respectively (P = 0.9). Similarly, the MDRD equation did not yield significant differences in cisplatin-eligibility.

**Figure 2:**
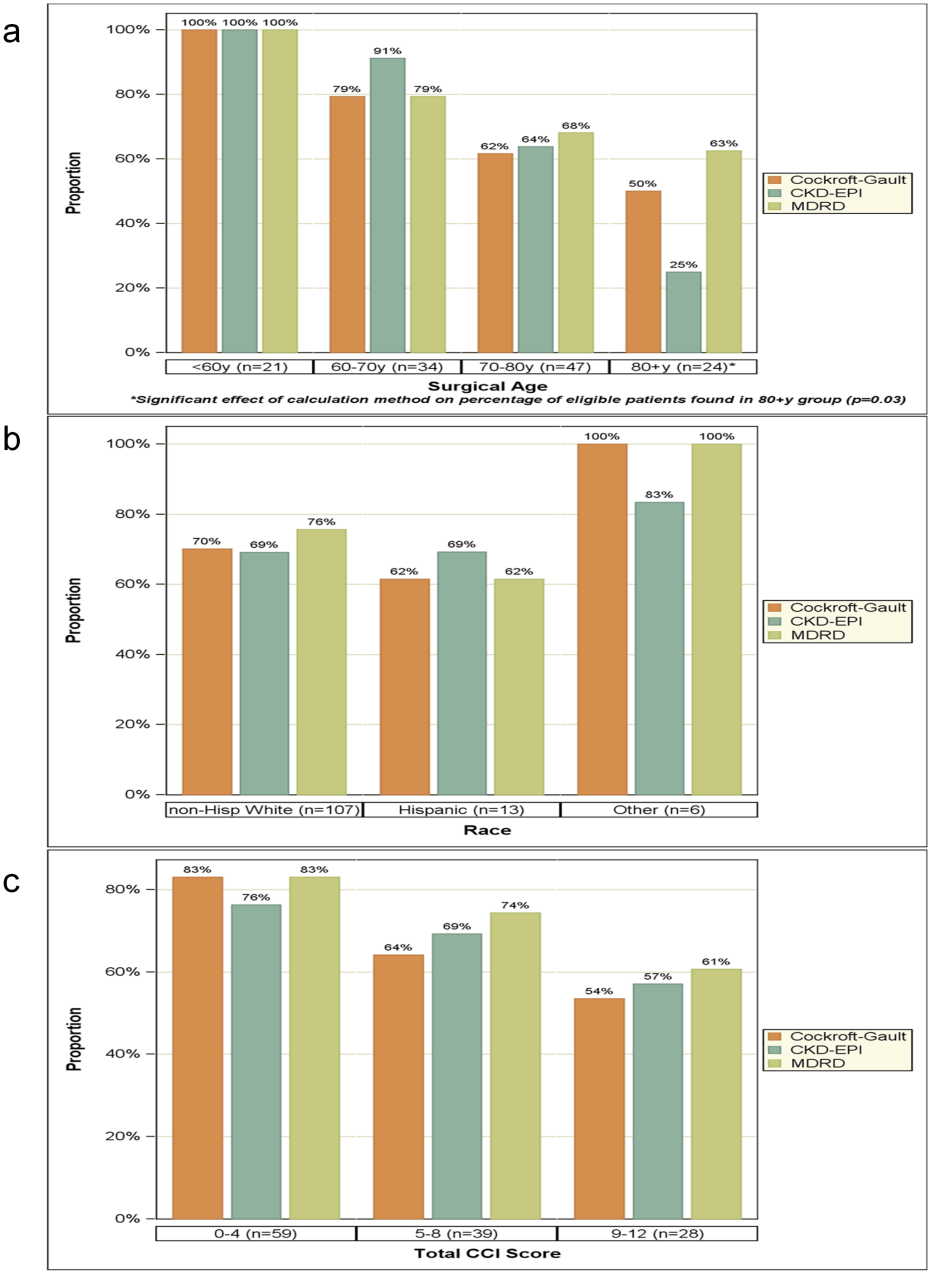
Proportion of patients defined as cisplatin-eligible (i.e., creatinine clearance >60) based on the CKD-EPI equation, Cockroft-Gault equation, and MDRD equation in subsets based on age (a), race (b) and comorbidity (c).

Analyses were subsequently performed, assessing cisplatin eligibility using the same three equations in subsets divided by age, race and CCI score. No differences in the proportion of cisplatin-eligibility were seen in patients <60, 60–70 or 70–80 years of age (Figure 2a). However, in the subset of patients age >80 (n = 24), the CKD-EPI equation identified a much smaller proportion of cisplatin-eligible patients (25%) as compared to the Cockcroft-Gault equation (50%) or the MDRD equation (63%). By race, the three equations yielded few differences in cisplatin-eligibility amongst patients characterized as non-Hispanic white, Hispanic white, and other (Figure 2b). Notably, our study included only 1 black patient, limiting our ability to infer the concordance of the 3 equations in this racial group. No significant differences in cisplatin-eligibility were noted in subsets divided by CCI score, irrespective of the CrCl equation used (Figure 2c).

## Discussion

In the current study, we could not replicate the results reported previously by Tsao *et al*.[16] Specifically, we did not find that the CKD-EPI equation was more likely to deem patients cisplatin-eligible as compared to the Cockcroft-Gault equation. On the contrary, our findings suggest that the CKD-EPI equation may be less likely to deem patients cisplatin-eligible in patients age >80. Similar findings were noted in the subset of patients defined as “other” race.

As also acknowledged by Tsao *et al*, reports such as these can only be characterized as hypothesis generating. However, we do take the added step of characterizing actuarial use of neoadjuvant chemotherapy in our cohort, which was comprised entirely of patients with MIBC (Tsao *et al* assessed a mixed cohort of patients with both localized and metastatic disease). As noted in Table 1, of 95 patients with a CrCl >60 mL/min by Cockcroft-Gault, only 31 patients (33%) received this modality. These data suggest that even if the CKD-EPI equation resulted in a modest increase in estimated CrCl, many patients with satisfactory values would still not receive neoadjuvant chemotherapy. Other large series have produced similar findings. For instance, amongst 238 patients with MIBC receiving cystectomy at the University of Texas Southwestern Medical Center, it was noted that 97 patients (67%) had renal function adequate for cisplatin.[17] However, only 25 patients (17%) ultimately received neoadjuvant chemotherapy. A comparison of recipients and non-recipients of neoadjuvant chemotherapy with a CrCl >60 mL/min in our series shows little difference in baseline characteristics (e.g., BMI, CCI and clinical stage) between the groups. It is worth noting that there are other objective factors outside of CrCl that may impact use of neoadjuvant chemotherapy, such as neuropathy, hearing loss, performance status and comorbid conditions – certainly, these factors may limit use of neoadjuvant chemotherapy in a small proportion of patients. However, we envision that other subjective factors (patient preference, physician preference, etc.) may play a larger role in guiding the decision regarding neoadjuvant therapy.

Several limitations of the study should be noted. First, comparisons are offered between three equations frequently used to estimate CrCl. However, none of these equations represents a “gold standard” – ideally, comparisons would be made to measured CrCl. It has been suggested that the measured CrCl may be the optimal means by which to discern appropriately discern candidates for cisplatin therapy.[18] Although time consuming and perhaps more costly, the measured CrCl would likely offer the most precise estimate of renal function. An alternative to measured CrCl might be the addition of serum cystatin C to a standard laboratory panel; the combination of serum cystatin C to estimated GFR by CKD-EPI seemed to predict actual GFR with greater precision.[19] A second limitation is that the data presented herein is derived from a single institution. A larger sample derived from multiple institutions may provide a more realistic account of practice patterns for MIBC. As noted, only 17% of patients who were cisplatin-eligible (based on a Cockcroft-Gault estimated CrCl >60 mL/min) received neoadjuvant treatment. These numbers are lower than estimates of neoadjuvant chemotherapy use from the National Cancer Database published in 2007.[20] A third notable limitation is that we omit salient clinical endpoints from our analysis, such as disease-free survival (DFS) or overall survival (OS). We have previously published outcomes of patients receiving neoadjuvant CG and MVAC from our institutional series in a separate report.[21] Ultimately, it would be informative to know if misclassification of patients as cisplatin-ineligible has a detrimental effect on clinical outcome. However, as the data herein suggests, there are multiple factors that may ultimately dissuade use of neoadjuvant chemotherapy. Clearly, cisplatin “eligibility” based on renal function is not the only driver of neoadjuvant chemotherapy use. An additional limitation of our work is that the primary reason for non-receipt of neoadjuvant chemotherapy (e.g., patient preference, physician preference, renal function, etc.) was not consistently documented amongst our patients – collecting this data retrospectively would be challenging and subject to substantial biases. Finally, we have not reported receipt of adjuvant chemotherapy. The role of adjuvant chemotherapy is debatable, and many key trials of adjuvant treatment have been plagued by methodologic issues and problems with accrual that preclude useful results. Furthermore, given that we are a tertiary care center, many patients receive their preoperative and operative treatment at our site but return to a local practitioner for further care. As such, our capture of receipt of adjuvant therapy may be incomplete.

Galsky *et al* have proposed a consensus definition to identify patients with metastatic urothelial carcinoma “unfit” for cisplatin chemotherapy.[10], [22] The definition is based in part on renal function, either measured or estimated. If estimated, a suggestion is made that the CKD-EPI equation be used, referencing a pooled analysis comparing the MDRD and CKD-EPI in non-oncology populations.[15] Although our study has the previously noted limitations, we reflect on the relative merits of the Cockroft-Gault, MDRD and CKD-EPI equations in a population solely comprised of patients with MIBC. Based on our data, it may be premature to substitute the CKD-EPI equation for the Cockroft-Gault equation in forthcoming trials in bladder cancer evaluating cisplatin-based neoadjuvant chemotherapy. To err on the side of caution, these studies may default to using measured CrCl to determine eligibility. Although a trial specifically dedicated evaluating the aforementioned equations is unlikely to occur, a prospective comparison of these equations could easily be embedded in any forthcoming study assessing neoadjuvant cisplatin-based chemotherapy.
